# Survey data of foreign language learners' enjoyment and anxiety in the U.S.

**DOI:** 10.1016/j.dib.2020.105221

**Published:** 2020-01-31

**Authors:** Tsung-han Weng

**Affiliations:** University of Kansas, Department of Curriculum and Teaching, United States

**Keywords:** Foreign language learning, Emotions, Enjoyment, Anxiety, Positive psychology

## Abstract

The data derive from a survey collected from 182 bilingual and multilingual speakers who are foreign language speakers from six language groups, including Mandarin Chinese, Japanese, Korean, Arabic, French, and Russian. They registered in beginning, immediate, and advanced levels of foreign language classes in a public four-year university in the Midwestern U.S. The survey was developed with an aim of exploring foreign language learners' enjoyment and anxiety in learning foreign languages. The survey was distributed by utilizing an online questionnaire, which is composed of four sections: 1) demographic information (9 items), 2) the Foreign Language Enjoyment Scale (FLES) (12 items), 3) the Foreign Language Classroom Anxiety Scale (FLCAS) (8 items), and 4) open-ended questions (2 items). Both FLES and FLCAS took the form of a 5-point Likert scale. The entire dataset is stored in an Excel file (.xls). The entire questionnaire is included as a supplementary file.

**Table:** Specifications Table

Subject	Education
Specific subject area	Foreign language learning, emotion, enjoyment, anxiety
Type of data	Excel files
How data were acquired	Survey (Qualtrics): https://kusurvey.ca1.qualtrics.com/jfe/form/SV_a924B7wzbGvzQln
Data format	Raw
Parameters for data collection	The survey targeted university foreign language learners in the U.S.
Description of data collection	The sampling is twofold: 1) invitation emails were sent by the language coordinators to all students who are currently enrolled in foreign language classes; and 2) the researcher contacted foreign language instructors to gain permission to visit classes and allow their students to complete the questionnaire.
Data source location	Numerous foreign language classes in a public four-year university in the Midwestern U.S.
Data accessibility	With the article

**Table:** 

**Value of the Data** • The data represent one of the first and largest samples in exploring foreign language learners' emotions in the U.S. • The data can be compared across different second or foreign language learning contexts in the world for further insight • The data can be examined the relationship between enjoyment and anxiety in foreign language learning • The data can be further statistically analysed to examine the links between enjoyment and anxiety and learner-internal variables and teacher-related variables • The data are beneficial for second or foreign language researchers who are interested in exploring foreign language emotions

## 1 Data description

The data derive from the results of a questionnaire to explore learners' enjoyment and anxiety in learning foreign languages at a Mid-western U.S. university. The data were collected from the self-report FLES and FLCAS to assess foreign language learners' perceptions, feelings, and attitudes of their enjoyment and anxiety. The excel sheet (.xls) contains raw data from the 52 Likert-scale items. The data contain nominal, ordinal, and continuous variables. Item 1 to 9 are participants' demographic information, including their gender, age, and race and ethnicity. Item 10 is participants' overall attitude towards foreign languages. Item 11 to 20 are participants' perceptions toward lecture-based teachers. Item 21 to 30 are participants' perceptions towards drill-based teachers. Item 31 to 42 are participants' perceptions on foreign language enjoyment. The scale has been tested to ensure its validity and reliability, with its global FLE Cronbach's *α* reliability coefficient 0.86, FLE-Social 0.87, and FLE-Private .78. Item 43 to 50 are participants' anxiety in foreign language learning. The Foreign Language Classroom Anxiety scale (FLCAS) developed by Horwitz et al. was used to measure students' level of anxiety in foreign language classrooms. The original scale consists of 33 items. Following Dewaele [1] and MacIntyre (2014), this dataset extracted 8 items from Horwitz et al's (1986) scale by focusing on items about physical anxiety. Item 51 and 52 provide participants' qualitative data on foreign language enjoyment and anxiety. Following Dewaele and Maclntyre, this dataset included open-ended questions at the end the questionnaire. The questions asked participants to recall their specific event in the classrooms in which they enjoyed or suffered from anxiety.

## 2 Experimental design, materials, and methods

Upon IRB approval from the Undergraduate Programs, participants were recruited through an invitation email sent by the language coordinators to all students who are currently enrolled in foreign language classes regardless of their age, genders, ethnic group, or nationalities. Participants completed an online consent form and questionnaire accessible through an email sent to their school email accounts. We also contacted foreign language instructors to gain permission to visit classes and allow their students to complete the questionnaire. We distributed flyers to students who were interested in participating in this research project and answered any questions potential participants might have about the project. On the flyer, it entails the research procedure: connect to internet, scan the QR code, and fill out the questionnaire. The whole procedure took approximately 7 minutes. The survey was administered in November and December in 2019.
