# Supplementary material for: Survey data of foreign language learners' enjoyment and anxiety in the U.S
Source: Data Brief. 2020 Jan 31;30:105221. doi: 10.1016/j.dib.2020.105221 (PMC7139155; doi:10.1016/j.dib.2020.105221)
Supplement: Multimedia component 2 [file mmc2.docx]

A research team in the Department of Curriculum & Teaching is interested in knowing more about your language learning experiences this semester! Tsung-Han Weng and Kristen Cypret are doctoral students in Curriculum & Teaching, and they are part of a research team working to understand more about students’ experiences of foreign language enjoyment and anxiety in the language classroom. They would like to invite you to participate in the 5 to 8-minute online questionnaire. Also, if you are interested in participating in a short focus-group interview (10-15 minutes), you’ll be able to provide an email at the end of the survey.

Thank you for your consideration!

To access the questionnaire, please take a picture of the following in a phone app or follow the link below.


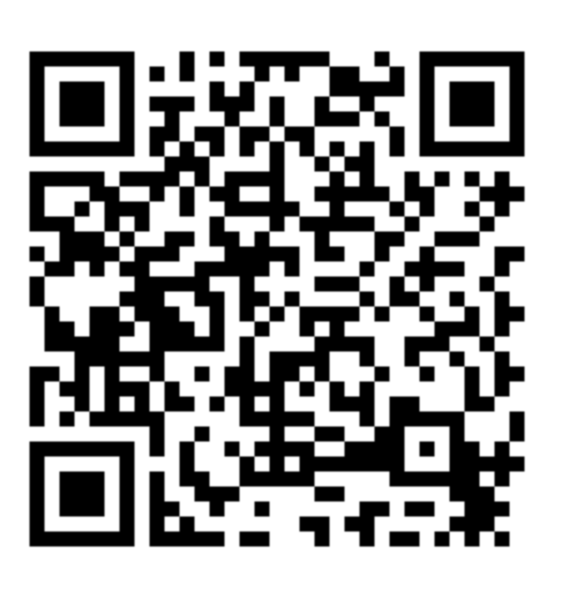


<https://kusurvey.ca1.qualtrics.com/jfe/form/SV_a924B7wzbGvzQln>

contact information: Tsung-han Weng [thweng@ku.edu](mailto:thweng@ku.edu) & Kristen Cypret [k563c011@ku.edu](mailto:k563c011@ku.edu)
